# Appropriating and asserting power on inflammatory arthritis teams: A social network perspective

**DOI:** 10.1111/hex.13051

**Published:** 2020-03-17

**Authors:** Wendy Hartford, Catherine Backman, Linda C. Li, Annette McKinnon, Laura Nimmon

**Affiliations:** ^1^ Department of Occupational Science and Occupational Therapy Faculty of Medicine University of British Columbia Vancouver BC Canada; ^2^ Department of Physical Therapy Faculty of Medicine University of British Columbia Vancouver BC Canada; ^3^ Arthritis Research Canada's Patient Advisory Board Vancouver BC Canada; ^4^ Centre for Health Education Scholarship Faculty of Medicine University of British Columbia Vancouver BC Canada

**Keywords:** agency, control, identity, illness stories, inflammatory arthritis, power, treatment decisions

## Abstract

**Background:**

Therapeutic interventions for people with inflammatory arthritis (IA) increasingly involve multidisciplinary teams and strive to foster patient‐centred care and shared decision making. Participation in health‐care decisions requires patients to assert themselves and negotiate power in encounters with clinicians; however, clinical contexts often afford less authority for patients than clinicians. This disadvantage may inhibit patients' involvement in their own health care.

**Objective:**

To identify communication attributes, IA patients use to influence and negotiate their treatment with members of their health‐care network.

**Method:**

A qualitative social network approach was used to analyse data from a larger study that investigated IA patients' overall experiences of multidisciplinary care. Fourteen patients with IA attended individual semi‐structured interviews. Researchers used thematic analysis to identify patterns of assertiveness and influence in the data.

**Results:**

Participants experienced loss of identity, control and agency in addition to the physical symptoms of IA. However, they had a sense of personal responsibility for managing their health care. Perceptions of health‐care team support enhanced patients' influence in treatment negotiations. Notably, there appeared to be an underlying tension between being empowered or disempowered.

**Discussion and conclusions:**

The findings have significant implications for treatment decision communication approaches to IA care. A social network perspective may provide a pathway for clinicians to better understand the complexities of communication with their patients. This approach may reduce unequal power dynamics that occur within clinician/patient interactions and afford people with IA agency, control and affirmation of identity within their health‐care network.

## 1 INTRODUCTION

Therapeutic interventions for people with inflammatory arthritis (IA) increasingly involve multidisciplinary teams with a focus on patient‐centred care and shared decision making.^1^, ^2^, ^3^, ^4^, ^5^ A typical approach to IA care (Box 1) acknowledges patients' rights to be involved in their own health‐care decisions,^8^, ^9^, ^10^, ^11^ appears to limit consequences of IA and contributes to better outcomes for patients.^2^, ^3^, ^4^, ^5^, ^9^ However, studies illuminate how the ideals of patient‐centred care^5^, ^12^, ^13^ and shared decision making^11^, ^14^ do not guarantee patients' involvement in their health‐care decisions. There is considerable variability in people's desire and ability to participate.^5^, ^11^, ^12^, ^15^, ^16^ Furthermore, the level of desire and ability to participate may change or fluctuate over time as people's physical and psychological distress increases or decreases^17^, ^18^; they learn more about their disease^5^, ^15^, ^17^; their disease progresses or stabilizes^11^; or their priorities shift across the life course.^5^

BOX 1 **IA Multidisciplinary care pathway**

The standard care pathway for inflammatory arthritis to reduce symptoms and limit joint damage and disability requires early identification and rheumatology referral,^2^, ^3^ and multidisciplinary patient‐centred care with rehabilitation professional referral as appropriate for the patient.^3^ For example, current treatment recommendations for RA emphasize:

- Early, aggressive treatment and a ‘treat‐to‐target’ approach (T2T)^6^: the escalation of treatment until a target is reached which is modified when target is no longer being met^6^, ^7^
- Target is to achieve and maintain remission or low disease activity in cases of established long‐standing disease^6^, ^7^
- Medical treatment with non‐steroidal anti‐inflammatory drugs, disease modifying anti‐rheumatic drugs and biologics^2^, ^3^, ^6^, ^7^
- Management of established RA: most patients require long‐term drugs with follow‐up every 3‐6 months and then 6‐12 months after suppression
- Non‐medical treatment according to patients' specific needs^2^, ^3^
- Attention to psychological effects, chronic pain, deficiencies of the immune system, increased cardiovascular risk, comorbidities and osteoporosis^2^
- Patients are encouraged to participate in decision making and self‐management over the long term^3^
- Encourage smoking cessation and weight management

Individuals also need to exert control in their medical negotiations,^15^ but this may be difficult for patients.^15^, ^19^, ^20^ This is in part due to clinicians' institutionally legitimized expertize which affords them a position of power and control of the encounter and medical decision negotiations.^10^, ^16^, ^19^, ^20^, ^21^, ^22^ Power is the ability of an individual to attain one's goals^22^, ^23^ and describes important relational dynamics that shape patient/clinician relationships and patient health outcomes.^8^, ^10^, ^20^, ^21^, ^22^

Power imbalances in the clinician/patient relationship may place patients at a disadvantage and inhibit patients' active involvement in their own care.^9^, ^16^, ^24^ Patients who feel in control and actively participate in their health‐care decisions experience improved health outcomes and quality of life, and optimized health/disease management.^5^, ^9^, ^25^ Therefore, it is critical that patients are partners in their care and negotiate their treatment plans. To achieve this goal, power disparities between clinicians and patients need to be illuminated and addressed.^15^, ^16^, ^22^ IA is the most common cause of disability in Canada^1^ and requires long‐term management and adherence to treatment to minimize joint damage and reduce physiological and psychological symptoms.^5^ Variations in models of care^26^, ^27^ make IA an ideal condition to examine differences in patient experiences of team care. Research that illuminates how people with IA assert themselves in medical decision making is limited. In particular, there is a lack of empirical research that examines the concept of power in the IA patient/clinician medical encounter from the patient perspective.

We sought to specifically explore how patients, from their perspective, assert themselves in their care network, including whether they assert themselves similarly or differently with various health‐care team members. The goal of this research is to gain a broader and deeper understanding of how patients take control of their treatment negotiations within their care network and identify communication mechanisms patients use to assert themselves and influence their treatment decisions.

## 2 METHODS

This research, set within the Canadian health‐care context (Box 2), was part of a larger study that investigated patients' overall experiences of being diagnosed with inflammatory arthritis and their interactions with clinicians and other health‐care supporters. The study was approved by a research ethics review board.

BOX 2 **The Canadian health‐care context**

Canada has a universal health‐care scheme (Medicare) which provides access for all Canadian residents to medically necessary hospital and physician services. Twenty‐eight Medicare is federally funded through transfer payments to the provinces and territories.^28^ The federal government holds the provinces accountable to providing universal health care through the Canada Health Act. In British Columbia, publicly funded non‐physician health‐care services are administered by the health authorities.^29^ Rehab services provided by health authorities are covered under the Medical services Plan. Extended health‐care plans, which may fully or partially cover additional services, are available through employment schemes or as individuals. Individuals without extended health‐care pay in full for additional services. Pharmacy services are covered under the Pharmacare Program.

### 2.1 Study design

Drawing on data from a larger qualitative study that explored IA team‐based care, we sought to answer the research question ‘How do patients with inflammatory arthritis experience influence, authority, and control in their health‐care decision within their care network?’ A qualitative social network perspective guided the larger study design and subanalysis of data that we report here. A social network paradigm focuses on relationships between actors in a social network.^30^ Particular attention is given to individual characteristics and relationships (ties) that enable and/or constrain choice and agency.^30^ For instance, communication pathways are ties by which actors in networks interact and exchange knowledge, ideas, views and resources.^30^ Different social networks influence individuals' perspectives, values, ideas and knowledge.^30^ People attempt to establish and stabilize themselves within a social network by forming an identity that is part of a discourse community of a social context.^31^ A person's identity is a composite of identities formed by the various social contexts they have been, and are, exposed to.^30^ Typically, networks refer to individuals, teams or organizations.^30^ We specifically focused on the ego networks of people with IA. An ego network analysis involves exploring individuals' perceptions and attributes of their network relationships.^30^ When people are diagnosed with IA, their lives change which destabilizes their identity.^32^ This in‐depth analysis was aimed towards a nuanced understanding of patients' identity development, and control and agency within a multidisciplinary team. This may provide novel insights into the flow of power among members of patients' multidisciplinary teams and help us better understand affordances which promote patient control over obtaining their goals.

### 2.2 Recruitment

Data collection for the larger study employed purposeful sampling to recruit participants 18 years or older, living in a major western Canadian city and surrounding area, diagnosed with IA (rheumatoid arthritis, spondyloarthropathies and connective tissue disorders) within the previous 5 years, who could identify two or more health‐care providers as being on their health‐care team. Invitations to participate were posted at arthritis clinics and on social media forums. Potential participants responded directly to the PI (AA) who along with several research assistants (BB, CC, DD, EE, and FF) interviewed participants who met the inclusion criteria. The research team consisted of patients, health professionals and researchers with an interest in IA (Box 3). The rich professional, academic and lived experiences of the research team enriched the interview questions. The research teams' diverse perspectives coalesced into interview questions that captured various dimensions of the phenomenon we sought to explore.

BOX 3 **Research team background**

Research design team.

PI: A social scientist.

Health professionals: a rheumatologist, occupational therapist and physiotherapist.

Public participation: three people with IA.

Research interviewers for this subanalysis.

AA: the principal investigator is a social scientist.

BB: a rehabilitation science PhD candidate.

CC: an adult educator.

DD: an adult education MA student.

EE: Master of Occupational Therapy student.

FF: Master of Occupational Therapy Student.

The research design team and the majority of the interviewers were involved in data analysis. The authors of this paper include the social scientist, occupational therapist, physiotherapist, one of the public participators and the adult educator.

Prior to one‐on‐one interviews, arranged at participants' convenience, participants received a study consent package which explained the study and interview requirements. The interviewers used a social network survey and semi‐structured, patient‐centred interview protocol^33^ (Box 4) with open‐ended and probing questions. Adopting a patient‐centred approach,^34^ participants generated an ego network that depicted who *they* perceived to be on their multidisciplinary team. Participants were interviewed by phone or in person. Demographic data and field observations were collected at the time of the interview. The research team contacted participants at a later date to request an optional follow‐up interview to consolidate the data and explore questions that emerged from the primary interviews.

Primary and follow‐up interviews were approximately 50 minutes in length. Interviews were audio‐recorded, transcribed and anonymized. Participants were provided with the opportunity to review and comment on their interview transcripts.

BOX 4 **Examples of patient‐centred interview questions**

**Social network survey question**

As a guide for participants we outlined a health‐care team as including: (a) at least two health professionals working together (eg nurse, GP, specialist physicians, occupational therapist, social worker), and (b) the patient and (c) informal caregivers (eg family, friends, those who help around the home).

‘Who do you perceive as being on your healthcare team? List as many individuals as you like, they can be health professionals or informal caregivers.’

Participants were also asked to identify which members of their network had the most or least influence on their treatment decisions.

**Interview questions**

Do you ask for what you need around your care from the individuals you have described as providing you with care?

Do you ask questions, do you make your own health related decisions, and do you make requests?

Does the care you receive focus on your personal treatment goals and wishes?

Do you believe that you are responsible for managing your own arthritis care? If so, what is that like for you?

How do the people you've described use your experience, opinions, knowledge, and/or choices into your goals for care? Probe: Do you feel your voice is valued?

Do you see yourself as being a key member of your care team?

Do you see yourself as empowered or influential in terms of your health care decision making? Please explain.

Is there anything else you would like to add about your experiences? Please describe them.

**Follow‐up interview example question**

We are finding that participants have varying concepts of a health care team. Could you describe a supportive and well‐functioning health care team in terms of managing your IA? Where would you place yourself in this team?

### 2.3 Data analysis

We coded transcripts to describe phenomena around how patients perceived their authority to lead their own care within their network. This analysis followed a three‐stage iterative and cyclical systematic process: item analysis, pattern analysis and structural analysis. Item analysis involved compiling groups of similar items of interest (such as understanding IA, taking responsibility and ignoring advice) which led to the identification of primary codes for organizing the data. Pattern analysis involved a process of comparison, contrast and integration, organizing items together in higher‐order patterns: for instance, preparing for a consultation, expressions of influence and barriers to gaining power. As relationships were the unit of analysis, we looked for ties and relationships between the sets of patterns to generate structures. This structural analysis stage brought together pieces of an analytic puzzle to create an overall conceptually integrated picture of the phenomena under exploration.^35^ During structural analysis, we incorporated many initial codes and developed theoretically informed themes that drew on social network theory and were concerned with the interconnection between personal characteristics, network supports and social control. NVivo 11 software helped organize the data throughout analysis.

## 3 RESULTS

We recruited and interviewed 14 participants (Table 1): 12 female and 2 male, between the ages of 20 and 70 years diagnosed with rheumatoid arthritis = 7; ankylosing spondylitis = 3; psoriatic arthritis = 2; undetermined = 2. We conducted follow‐up interviews with 7 participants (2 male and 5 female).The following themes were generated from the analysis: personal responsibility, team support, and empowerment and disempowerment. Participants' descriptions of their health‐care network experiences revealed multiple ties which either facilitated or hindered their involvement in their treatment decisions. IA had dramatically altered aspects of participants' lives resulting in a sense of loss of agency, control and identity. Participants described loss of independence and previous functional ability associated with physical activity, work, household tasks, personal care, child care and shared social activities.

That was me through and through. I can do everything for myself. I didn't want to ask for help ever. And that was very much part of my identity and I would pride myself on that. And then, like, just really having to let that fall away, and that's so hard. That's a daily struggle. It's like I'm, like, oh, poor me, I want so much help, but also I don't want help, you know, and it's, like, those opposing tensions. (Jamie)

**TABLE 1:** Participant characteristics

Participant (age)	Gender	IA diagnosis	Health‐care team	Socioeconomic status
Amber (25)	Female	Ankylosing spondylitis 18 mo	Rheumatologist Physiotherapist GP MA supervisor Massage therapist Chiropractor Mom	Student, single, Extended health‐care plan^a^ Income $200 000 (parents)
Brenda (27)	Female	Psoriatic arthritis 18 mo	Rheumatologist Family physician Pharmacist Mom	Clerk administration 1 child Income $30 000
Cam (52)	Male	Rheumatoid arthritis 10 mo	Rheumatologist Physician Occupational therapists Physiotherapists Family and friends provide non‐medical support	Single Unemployed Annual income $5000
Danielle (39)	Female	Ankylosing spondylitis 2.5 y	Rheumatologist Family physician Intermittent help No real team support from online groups	Stay at home mom 2 children Extended health‐care plan^a^ Income NA
Erica (20) 6 mo	Female	Rheumatoid arthritis 6 mo	Rheumatologist Friends and family support and comfort	Student, single Income NA
Francois (62)	Female	IA undetermined 2 y	Family Physician Rheumatologist Husband Chiropractor Reflexologist Massage Therapist Medical Marijuana Dispensary	Retired, Government position Extended health care^a^ Income NA
Geoff (70)	Male	Rheumatoid arthritis 5 mo	Wife Rheumatologist Physician Physiotherapist	Retired firefighter Married, 1 adult child out of home Extended health care^a^ Income $125.000
Helen (33)	Female	Psoriatic arthritis 1.5 y	Rheumatologist Nurse Pharmacist Family Physician	Married 1 child Extended health care^a^ Income $100 000
Ingrid (59)	Female	Rheumatoid arthritis 5 y	Rheumatologist Neurologist Primary care doctor Family/friends Dermatologist OT Internist Immunologist ENT/audiology Urologist Home health care	Retired speech pathology teacher Single 2 adult children out of home Extended health care^a^ Income N/A
Jamie (31)	Female	Rheumatoid arthritis 12 mo	Rheumatologist Nurse Family physician Husband Mother	Clerical work Married, 1 child Extended health care^a^ Income N/A
Kathy (39)	Female	IA undetermined A few months	Counsellor GP Rheumatologist Physio Massage therapist	Small business selling cross stitch, on PWD (People with disability) Single, no children, Health‐care benefits through PWD
Lucy (32)	Female	Ankylosing spondylitis 4 y	Rheumatologist Physios (not current) Ophthalmologist Family physician	Stay at home mom Married 3 children Extended health care^a^ Income $90 000
Marie (32)	Female	Rheumatoid arthritis 2 y	Rheumatologist Spouse	Environmental health officer Married Extended health care^a^ Income $160 000
Nadine (32)	Female	Rheumatoid arthritis 2.5 y	Rheumatologist Family physician Pain Specialist Psychologist Spouse	Meteorologist Married Extended health care^a^ Income $130 000

^a^ All participants received basic health care under the universal healthcare plan available in the province. Extended health care refers to additional healthcare insurance offered by some employers to their employees.

Participants also described various communication attributes (Table 2) and nuanced ways (ties) which enabled them to position themselves within their health‐care network and thus gain agency and influence over their treatment decisions. Taking personal responsibility for their health and well‐being, and support from their health‐care network were important factors for gaining agency and control within their network. Descriptions also provided insights into participants' identities as people with IA.

**TABLE 2:** Summary of communication attributes

Communication attribute	Description of attribute
Taking responsibility for self	Working hard to improve health Showing an interest in health care
Self‐advocacy	Getting answers to questions Pushing for stronger medications
Being assertive	Not afraid to ask Push to be heard Don't quit Outspoken about health
Prepared for consultation to ask specific questions	Gathering appropriate IA information Compiling test results Tailor interactions to meet clinicians' personalities Understanding medical terminology
Switching clinician	Result of deteriorating relationship Lack of clinician empathy Preferences and illness experiences ignored
Using another clinicians opinion	To get perceived health‐care needs met: referral
Remaining silent	To avoid upsetting clinician, lose credibility, or lose clinician services
Resisting clinician advice	Not taking medication or rest as advised

### 3.1 Personal responsibility

Personal responsibility for disease management meant taking their health into their own hands, working hard to improve their health, showing an interest in their care, taking their medications and following clinician instructions.

I mean, it's up to me, you know, a lot of stuff is up to me. Whether I try hard to, you know, in physio or just take my medications as prescribed and doing all that....so I feel like that's important. (Cam)

Self‐advocacy was perceived as part of participants' network role. In itself, self‐advocacy could be empowering and provide the impetus to challenge proposed medical therapies.

When you have the energy to advocate for yourself it can be very empowering. Because you feel, at least, like, all your questions are being answered, you're not being left in the dark. You can keep track of your medications and know how those medications are going to help you. And then push for the stronger medications if things aren't working which is what I had to do. (Nadine)

Attributes such as being assertive, consultation preparation and use of medical terminology enabled participants to position themselves in their health‐care network. Participants described their ‘fight’ to be heard and get the treatment they perceived they needed. Other expressions of assertion included ‘I'm not afraid to phone and ask people’, ‘I push when people aren't listening’, having a ‘spirit of don't quit’ and being ‘outspoken about my own health’. Participants intentionally strategized and prepared for consultations with their physicians to ensure their voices were heard and to make the best use of the ‘brief’ time allotted to them. Preparation included gathering information about their diagnosis and potential treatment options from clinicians and the Internet, and compiling test results for their rheumatologists. This enabled participants to prepare appropriate, direct and specific questions for their consultation and to confidently engage in treatment decisions. One particularly challenging task was interacting with different clinician personalities:

So I have to be extremely strategic in what I want to cover…it depends on the doctor and one of the most challenging aspects of being a patient is tailoring my personal interactions with doctors to their particular idiosyncrasies and personalities. (Ingrid)

Consultation preparation also included understanding and using medical terminology as a way to influence treatment decisions. Using medical terminology helped some participants gain credibility and be taken seriously, and indicated they were interested in their own care. Medical terminology facilitated ease and accuracy of communication in treatment negotiations to ‘… be as accurate as possible. So that you can get the best advice back’. (Jamie)

However, tiredness, pain and depression negatively interfered with their ability to self‐advocate and assert themselves. Conversely, a tie that promoted participants' agency and control in treatment negotiations was clinician acknowledgement of and support for participants' goals.

### 3.2 Team support

The composition of participants' networks varied by number and professional/non‐professional identity. Several participants identified ties which supported and empowered them in treatment decisions with their various clinicians such as when their goals and values were acknowledged.

I think when it came down to me and [the Rheumatologist] discussing medications he was very good about informing me what each medication did and how it might benefit me and how it might not. And I felt like I had some say as to what I wanted to try, and when something didn't work he was very good at finding something else for me to try. And just like letting me stop the medication that wasn't working. He has never forced me to do anything I didn't want to do to help it. (Brenda)

Participants' unique health goals contributed to a varied social landscape of team support. While some participants received uncontested support for their goals and or felt supported by all members of their team, not all team members took participants' goals into consideration. Participants could experience differential support with their team members. However, team support could help participants regain their identity as a person:

And then over time with the physiotherapist and the occupational therapist and even kind of seeing like the counselor a bit, trying to work towards, you know, like, enjoying life and empowering and figuring out, like, you know, how to still be a person and not just a patient, I guess, with rheumatoid arthritis. (Marie)

When communication pathways appeared blocked, participants perceived they needed to take a stand or relinquish agency in their health‐care decision negotiation. Although not always explicit, participants' descriptions hinted at a tension between being empowered or disempowered.

### 3.3 The empowerment/disempowerment dynamic

The act of listening represented another tie which facilitated participants' agency and inclusion in treatment decisions. Clinicians who took time to listen to participants were perceived as supportive and provided an environment in which participants were able to express their goals and values, whereas experiences of not being heard, listened to, believed and taken seriously appeared to block participants' agency in attaining their treatment goals. For example, some participants reported clinicians assuming reported symptoms to be associated with participants' pregnancies and ignoring requests for investigative tests. Gender, which could contribute to being labelled non‐compliant, was also perceived as a barrier to effective communication. ‘And sometimes I think that it was because I was a woman that they never ever believed me’. (Lucy).

Nonetheless, these potentially disempowering situations, which constrained participants' agency in their health‐care network, did not render participants without some control. Participants identified various tactics they used to establish control to have their needs met. When faced with deteriorating clinician relationships, participants took control of the situation by switching clinicians. Lack of compassion when first diagnosed, desiring a different approach to treatment, perceptions of symptom dismissal and of disbelief, and failure of clinicians to meet their needs were all reasons for finding a new clinician. However, clinicians were also found to fracture relational ties with patients when patients challenged clinicians' medical authority: for example. ‘But I'm saying as the patient I don't think I have what you‐‐ I've looked up what this disease is and I don't think I have that one…that got me dismissed really quickly.’ (Francois).

Actions patients used to have their treatment needs met included seeking second specialist opinions, challenging a clinician and using the influence of another clinician to support their request.

My rheumatologist said that this could be IBD and it's associated with ankylosing spondylitis …and he thought that I should probably see a GI specialist. Pretty sure I got, like, a look of death from my GP in that moment. He was not happy but he said, ‘Okay, I'll refer you to someone’. So he referred me to someone. (Amber)

However, such interactions could be counterproductive and result in loss of agency and control and fractured communication: ‘… as soon as he kind of got angry about that situation, it made me very much not want to talk to him about anything else in that appointment. It felt like I stepped on some toes’. (Amber).

To avoid rupturing relational ties with clinicians and potentially losing clinicians from their team, participants purposely remained silent and followed advice to avoid ‘rocking the boat’, adversely influencing their treatment plan or losing credibility. In one instance, a participant ‘got a kick’ out of keeping quiet about his knowledge.

Participants refrained from disclosing information which they perceived might not be favourably received by a specific clinician such as a fibromyalgia diagnosis, and psychiatric treatment or naturopathic treatment. ‘Tamping down’ and testing how much to share with the team was another strategy to avoid fracturing relationships. ‘I knew what I had at risk because these professionals have a lot of power over my care and my treatment…the equal partner in care concept at this point in time is a joke’. (Ingrid).

Flow of information along communication pathways could also be blocked when physicians were not open to alternative information diagnosis, medication options or drug side‐effects. Physicians may discourage and/or discredit patients who independently seek information from sources other than their health‐care provider. Participants described defying advice to take prescribed medications and to rest. However, these expressions of agency and control, at times unknown to other team members, did not appear to fracture relational ties between participants and their clinicians. Overall, there was an implicit dynamic between being empowered and disempowered which appeared to reflect participants' perception of their position and identity within their health‐care network. For instance, Geoff, despite considering himself empowered, described himself as a *‘subservient’*, but stoic, member of his team who tried to, ‘*but not always*’, co‐operate with the experts who ‘*know what they are doing*’. While self‐knowledge appeared to support participants' perceptions of influence in IA care management, this did not translate to a sense of holding equal power when negotiating with clinicians: ‘…actually as a patient I have no power…. really no say’. (Danielle).

## 4 DISCUSSION

This social network study contributes to a scarce conversation in the literature that explores how IA patients assert themselves and gain control in treatment negotiations with their health‐care network. Specifically, this work highlights the significance of open communication pathways which acknowledge patients' experiential expertize as valuable forms of knowledge in treatment decisions. Our analysis indicated participants, who considered themselves to be integral to their own network, wanted to be active partners in their treatment negotiations. This sample thus described a high degree of patient activation. The concept of patient activation alludes to a patient's knowledge, skill and confidence in self‐managing their care and their ability to acknowledge and fulfil their active health management role.^36^ According to previous literature, higher levels of patient activation may contribute to greater patient control of their health care and contribute to better health outcomes^36^ However, there was an inherent contradiction that emerged in our analysis as participants' seemingly high degree of patient activation did not necessarily provide them with greater control or influence in their treatment decisions. Mirrored by a perspective offered by Pratto,^23^ having agency, the ability to act, did not ensure that these participants had power: they faced barriers to achieve their health‐care goals and needs.

This analysis suggests people with IA seek control of their treatment negotiations through agency and establishing identity. An important contribution of this analysis is that acknowledgement of people's illness accounts may be necessary to promote the flow of power through communication pathways.

### 4.1 Seeking control through agency and establishing identity

Findings revealed personal characteristics and communication attributes where participants attempted to gain agency, identity and exert control in their treatment negotiations. Chronic illness destabilizes a person's identity and sense of self.^37^, ^38^, ^39^ People naturally seek to reconstruct their identity within their new social context by seeking control to establish their position within a network.^31^ Our findings, similar to the literature, suggest patients with chronic inflammatory conditions invest time, work hard to meet their health‐care needs,^38^, ^40^ show an interest in their care^17^, ^38^, ^40^ and consider it their responsibility to actively participate in their treatment decisions.^5^, ^25^ Assertiveness promotes engagement in decision making^11^ and may disrupt^16^ and inhibit^24^ clinicians' attempts of control.

Knowledge acquisition, a core component of an individual's illness identity,^16^ enables patients to exert control in their treatment negotiations.^12^, ^16^ Knowledge and its relationship to power is intrinsic to patient/clinician encounters.^12^, ^16^ However, our findings confirm knowledgeable, questioning and agentic patients are sometimes met with opposition. This may result in patients being labelled as difficult^9^, ^12^, ^16^, ^41^ and non‐compliant.^12^, ^16^, ^39^ In some instances, physicians may denigrate patients for their learning^39^ and be dismissive.^16^ From a social network perspective, people's views and beliefs can influence the behaviour of other individuals^30^ which our analysis suggests may constrain patient agency and the flow of power.

Patients who fear being perceived as difficult, potentially creating conflict and jeopardizing treatment,^15^, ^20^ may endeavour to be perceived as a ‘good’ (normative) patient who refrains from challenging medical authority.^15^, ^16^ Our findings imply that participants developed various strategies to be perceived as credible and/or good patients^38^ and used different strategies at different points in time to achieve their goals. Socially internalized norms, a good or difficult patient for example, may not assign authority and can thus constrain patient agency.^39^ However, conforming to these norms and deferring to clinician authority does not necessarily remove patient agency, identity and control.^16^, ^23^ Rather, it was sometimes a way of exerting control.

Agency can be communicated verbally and non‐verbally.^31^ As reported in our findings, patients may not adhere to their physician's prescribed treatment^8^, ^12^, ^16^, ^21^ and may discard prescriptions and change physicians.^8^ However, as previously identified in the literature,^8^, ^12^, ^16^ participants' challenges were not well accepted by health‐care providers. Taking a social network perspective, discordance within a group may reflect differences, or perceptions of differences, in individuals' structural positions within their network. Members of a network have characteristics, such as expertize, which place them in various structural positions within the network.^30^ Such characteristics can be considered symbolic capital which, when recognized by a group, legitimizes that individual's power.^41^, ^42^, ^43^ Thus, the structural position of an individual can determine network outcomes,^30^ in this instance the flow of control and power.

Admonitory and censorious non‐lingual expressions of power,^31^, ^42^ such as the ‘look of death’, may inhibit further treatment discussion and negotiation. Language, particularly when tied to structures of power such as the social institution of medicine, can be a vehicle for the expression of power.^10^, ^16^, ^41^, ^42^ However, as our findings suggest, patients rebel against such expressions of power by selectively using medical terminology,^12^, ^16^ remaining silent and holding back information: recognized strategies for resisting authority, and gaining agency and control.^16^, ^42^ Although the power dynamic may not change,^23^ these concealed expressions are not passive or acquiescent, but instances of everyday resistance to perceived authority and evidence of control.^31^

### 4.2 Unheard and dismissed illness accounts

Stories, or accounts, can be considered ties through which individuals integrate their experiences from their various social networks.^31^ Recounting illness experiences helps individuals understand^21^, ^39^, ^44^ and promotes discussion of their illness experiences, enables identity development^31^, ^37^, ^44^ and provides a way for individuals to express power and be recognized as an autonomous person.^45^ Significantly, our analysis suggests that not being heard or believed rendered some participants powerless to gain agency and control in their treatment decisions. Not being heard,^21^, ^46^ listened to,^11^, ^15^ believed^40^, ^46^ or taken seriously^11^, ^40^, ^46^ are barriers to patient involvement in treatment negotiations. Such communication practices, which fracture communication ties, can lead to tension in the clinician/patient relationship,^21^ challenge women patients' self‐esteem and self‐worth when accounts of pain are disbelieved^38^, ^46^ and enable clinicians to exert control in the decision‐making process.^15^, ^24^ Decision practices that limit patients' involvement and autonomy fail to respect the patient's personhood^43^ and disempower patients which may constrain disease self‐management.^24^ Conversely, listening to, honouring and respecting people's illness accounts acknowledges their experiential expertize and promotes letting go of expert authority, power and knowledge.^37^, ^44^, ^45^

This analysis implies that for patients to engage equitably in treatment decisions, they need to be able to speak freely, without fear of reprisal, knowing that their voiced illness experiences will be acknowledged and respected.

### 4.3 Practice implications

A significant implication of this analysis concerns the role of communication in treatment decisions between patients and members of their IA multidisciplinary care teams. Communication provides a pathway for the flow of interactions, ideas, views and resources between people.^30^ From a social network perspective, Nimmon and Regehr^41^ suggest that current understanding of clinician communication may be insufficient to meet the demands of the complex communication ties that exist within patients' social networks. Focusing on building patient skills, knowledge and self‐management ability may not be sufficient to enable patients to be partners in their treatment decisions. We propose that a social network perspective to shared decision making, which emphasizes the importance of recognizing patients' experiential expertise as valuable knowledge, may provide a pathway for clinicians to engage patients as active partners in their treatment decisions. Specifically, and when framed within the context of a broad social ecosystem of care, a pivoting to patients' expertize that is lived and situated may redistribute hierarchical influence in decision making.

Just as clinicians' medical knowledge and experience may be considered symbolic capital which legitimizes their position of power, Locock et al^43^ suggest that patients' illness expertize should also be recognized as symbolic capital through which they can influence others. Patients' illness expertize refers to the reality of living with their illness and their ‘technical illness knowledge’ (p. 839).^43^ People with illnesses requiring long‐term self‐management develop illness knowledge that exceeds the accepted understanding of the illness.^43^ For example, arthritic joint pain is frequently accepted, erroneously, as part of normal ageing.^1^ When combined with a person's illness experience technical illness knowledge validates a person's symbolic capital potentially increasing their power and negotiating influence.^43^ However, professional and lay illness knowledge and expertize are not afforded equal recognition in medical decision processes.^9^, ^11^, ^15^, ^20^, ^43^ This is important to address since the flow of resources, such as power, through network ties is related to the level of actor control which is, in part, determined by their structural network position.^30^ Clinicians, however, may not be aware of the power differential and hierarchical assumptions that exist in their patient/clinician relationships,^10^, ^20^, ^24^ and the potential to promote or hinder patient/client agency in treatment negotiations.^22^, ^24^ It is important that clinician communication education explicitly address the concept of network structural position and flow of power (resources) through the network. Health educators need to draw on social network research in ways that can be applied to patient‐provider communication. Emphasizing patients' illness accounts as a pathway to foster ties through which power and identity can be co‐constructed may be critical if patients are to be agentic partners in their treatment decisions and gain control of their health‐care management. Symbolic capital without recognition of its value by members of a group cannot introduce a re‐imagining of influence in negotiations.^42^, ^43^ Therefore, it is important for network members to recognize and acknowledge the value of patients' experiential expertize as a mechanism to redistribute power. Although our focus here is on patients, we suggest experiential and professional expertize can be extended to cross‐disciplinary communication relationships within a patient's health‐care network. Introducing a responsive relational approach^47^ to multifaceted forms of expertize may improve communication ties and the flow of power within the network and enhance patient treatment decisions. A whole network analysis exploring communication mechanisms used by all members to assert themselves and influence patients' treatment decisions is an important area for future consideration.

Finally, ego networks are influenced by organizational structures which may control communication styles.^30^ We suggest that future research to support agentic patient participation in treatment decision making consider engaging a social network lens that can integrate insights from individual (ego), organizational (health‐care team) and institutional (policy) levels to refine understanding.

### 4.4 Limitations

The study findings are context‐specific and may not be transferable to other IA populations with different characteristics or lower levels of patient activation. Only two men participated which may reflect a selection bias and/or disease pathology. Of note, most forms of inflammatory arthritis affect more women than men. For instance, more than three of five people with rheumatoid arthritis are women.^48^, ^49^ Research also indicates that women may be more likely to self‐select for health research.^50^ Variation in structure of care networks limited our analysis with respect to identifying universal or varying power relationships between different network members. Future research which explores whether different care network structures are representative of differences in care and reflect different needs of people with IA may provide additional insight for multidisciplinary health‐care education. Future research could determine if there are differences related to age, gender or other sociodemographic characteristics. There was considerable variability in participants' ages yet our analysis did not identify any age‐related differences. Additionally, our analysis did not address perceptions of gender treatment inequality around experiences of pain which has been identified in the literature.^38^, ^46^ Furthermore, the data did not identify temporal changes in participants' perceptions of influencing treatment decisions.

## 5 CONCLUSION

We explored the experiences of people with IA to gain a broader and deeper understanding of communication mechanisms people use to take control over their treatment decisions. Through a social network lens, our findings suggest that despite loss of identity patients are agentic and have a sense of responsibility for their treatment decisions. Members of an individual's health‐care network should take into account the individual's lived experiential expertize because failing to do so may disempower patients and hinder participation in their treatment decisions. We suggest that members of patients' health‐care networks (health professionals and caregivers) could learn from social network approaches to develop a better understanding of social networks surrounding their patients. This approach fosters unconstrained respectful conversation wherein network members' knowledge and expertize, including that of the patient, is afforded equal importance as a resource for effective treatment negotiations. This relational approach to communication may reduce the unequal power dynamics that occur within clinician/patient interactions and afford people with IA agency, control and affirmation of identity within their complex health‐care network negotiations.

## CONFLICT OF INTEREST

No conflict of interest has been declared.

## AUTHORS CONTRIBUTIONS

Wendy Hartford collected, analysed and interpreted data, and drafted all components of the manuscript. Dr Laura Nimmon conceived of and designed the larger study and contributed to analysis and interpretation of data for the current paper. Dr Laura Nimmon was also involved in editing the manuscript and revising it critically for important intellectual content. Dr Catherine Backman, Dr Linda C Li and Ms Annette McKinnon contributed to analysis and interpretation of data and revising the manuscript critically for important intellectual content. All authors have given final approval of the version to be published and agreed to be accountable for all aspects of the work.

## ETHICAL APPROVAL

Ethics approval was obtained from the Behavioural Research Ethics Board (BREB), University of British Columbia (reference # H15‐01751).

## CONSENT TO PARTICIPATE

All participants provided signed consent prior to participating in the study.

## Data Availability

The data that support the findings of this study are available on request from the corresponding author. The data are not publicly available due to privacy or ethical restrictions.
